# The holiday season is over, but uric acid crystals party all year

**DOI:** 10.1590/2175-8239-JBN-2025-0003en

**Published:** 2025-08-22

**Authors:** Filipa Trigo, Rui Duarte, Paulo Santos

**Affiliations:** 1 Hospital de Torres Novas, Departamento de Nefrologia, Médio Tejo, Portugal.

**Figure 1:**
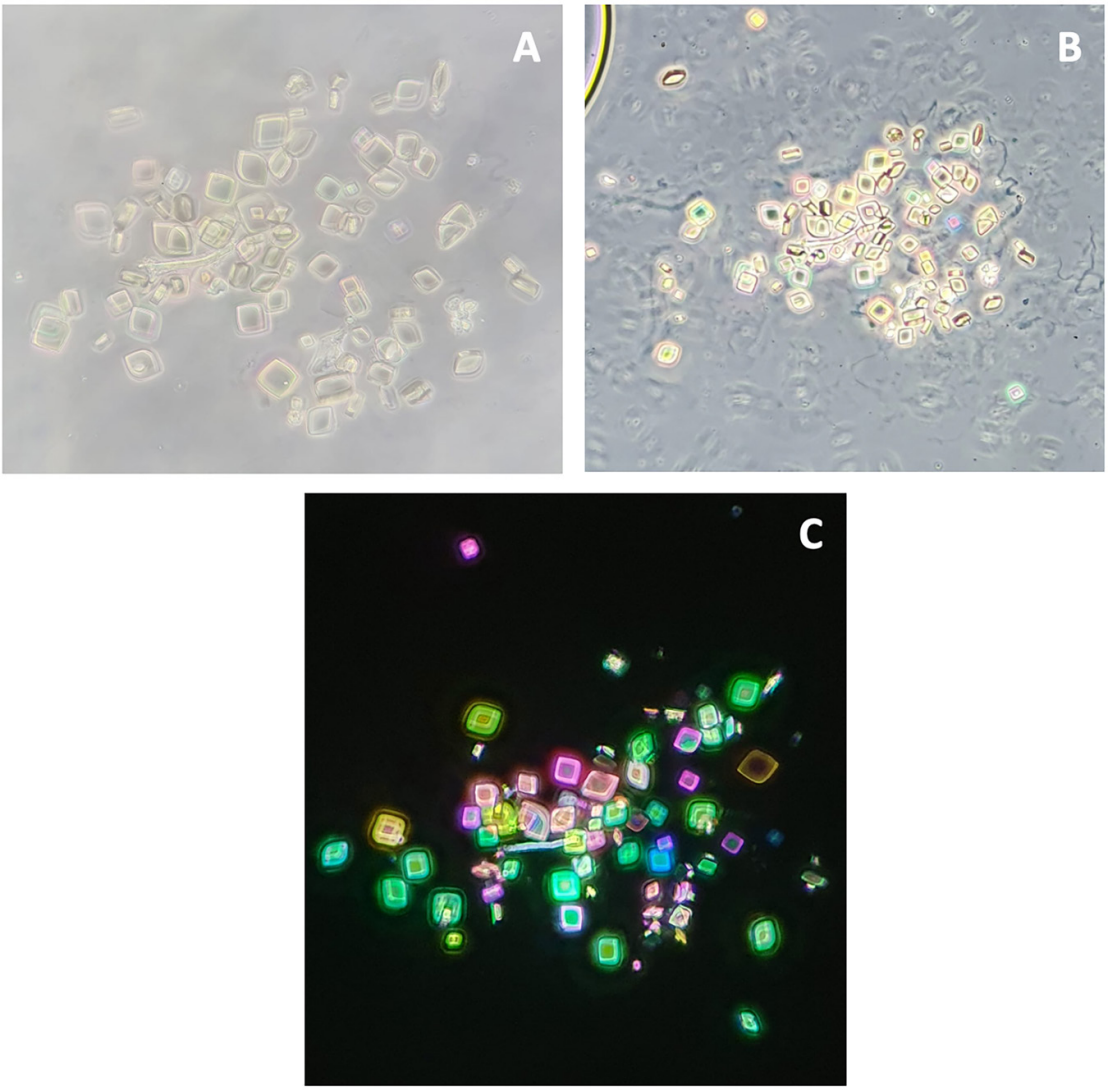
Images of urinary sediment with acid uric crystals - pleomorphic crystals under phase contrast (A and B) showing the same shape and different vibrant colors such as green, blue, orange, yellow and pink under polarized light (C).

This is a case report of a 42-year-old man with stage 4 chronic kidney disease (CKD) due to autosomal dominant polycystic kidney disease and a history of renal colic confirmed by stone excretion leading to clinical resolution; stone analysis was not possible. Sediment analysis revealed pleomorphic uric acid crystals (Figure 1 – A, B and C) forming in acidic urine (pH ≤ 5.5) and showing vivid birefringence under polarized light^1^.

Differential diagnoses include sulfame-thoxazole, xanthine and cystine crystals—distinguishable by clinical context and morphology. Febuxostat was initiated due to moderate renal dysfunction and the absence of cardiovascular disease. This case highlights the diagnostic value of urine sediment analysis in CKD, even when serum uric acid levels are normal^2^.
