# Supplementary material for: The holiday season is over, but uric acid crystals party all year
Source: J Bras Nefrol. 2025 Aug 22;47(4):e20250003. doi: 10.1590/2175-8239-JBN-2025-0003en (PMC12377895; doi:10.1590/2175-8239-JBN-2025-0003en)
Supplement: Translation

# Translation: As festividades terminaram, mas os cristais de ácido úrico festejam o ano todo

**DOI:** 10.1590/2175-8239-JBN-2025-0003pt

**Authors:** Filipa Trigo, Rui Duarte, Paulo Santos

**Affiliations:** 1 Hospital de Torres Novas, Departamento de Nefrologia, Médio Tejo, Portugal.

Trata-se de um relato de caso de um homem de 42 anos com doença renal crônica (DRC) estágio 4, decorrente de doença renal policística autossômica dominante e histórico de cólica renal confirmada pela excreção de cálculos, o que levou à resolução clínica. Não foi possível realizar a análise dos cálculos. A análise do sedimento urinário revelou a presença de cristais pleomórficos de ácido úrico (Figuras 1A, 1B e 1C), que se formaram em urina ácida (pH ≤ 5,5) e apresentaram birrefringência vívida à luz polarizada^1^.

**Figura 1:** Imagens de sedimento urinário com cristais de ácido úrico - cristais pleomórficos sob contraste de fase (A e B) exibindo a mesma forma e diferentes cores vibrantes como verde, azul, laranja, amarelo e rosa sob luz polarizada (C).

Os diagnósticos diferenciais incluem cristais de sulfametoxazol, xantina e cistina - distinguíveis pelo contexto clínico e pela morfologia. Febuxostate foi iniciado devido à disfunção renal moderada e à ausência de doença cardiovascular. Esse caso destaca o valor diagnóstico da análise do sedimento urinário na DRC, mesmo quando os níveis séricos de ácido úrico estão normais^2^.
